# Challenges and Opportunities in Digital Screening for Hypertension and Diabetes Among Community Groups of Older Adults in Vietnam: Mixed Methods Study

**DOI:** 10.2196/54127

**Published:** 2024-12-02

**Authors:** Trang Thi Thu Nong, Giang Hoang Nguyen, Alexander Lepe, Thuy Bich Tran, Lan Thi Phuong Nguyen, Jaap A R Koot

**Affiliations:** 1 HelpAge International in Vietnam Hanoi Vietnam; 2 Health Strategy and Policy Institute Vietnam Ministry of Health Hanoi Vietnam; 3 Department of Health Sciences University Medical Center Groningen University of Groningen Groningen Netherlands; 4 Faculty of Public Health Thai Nguyen University of Medicine and Pharmacy Thai Nguyen Vietnam

**Keywords:** NCD screening, DHIS2 tracker, District Health Information Software, version 2 tracker, digital application, ISHC health volunteers, non-communicable diseases, prevention, Vietnam, mobile phone

## Abstract

**Background:**

The project of scaling up noncommunicable disease (NCD) interventions in Southeast Asia aimed to strengthen the prevention and control of hypertension and diabetes, focusing on primary health care and community levels. In Vietnam, health volunteers who were members of the Intergenerational Self-Help Clubs (ISHCs) implemented community-based NCD screening and health promotion activities in communities. The ISHC health volunteers used an app based on District Health Information Software, version 2 (DHIS2) tracker (Society for Health Information Systems Programmes, India) to record details of participants during screening and other health activities.

**Objective:**

This study aimed to assess the strengths, barriers, and limitations of the NCD screening app used by the ISHC health volunteers on tablets and to provide recommendations for further scaling up.

**Methods:**

A mixed methods observational study with a convergent parallel design was performed. For the quantitative data analysis, 2 rounds of screening data collected from all 59 ISHCs were analyzed on completeness and quality. For the qualitative analysis, 2 rounds of evaluation of the screening app were completed. Focus group discussions with ISHC health volunteers and club management boards and in-depth interviews with members of the Association of the Elderly and Commune Health Station staff were performed.

**Results:**

In the quantitative analysis, data completeness of all 6704 screenings (n=3485 individuals) was very high. For anthropomorphic measurements, such as blood pressure, body weight, and abdominal circumference, less than 1% errors were found. The data on NCD risk factors were not adequately recorded in 1908 (29.5%) of the screenings. From the qualitative analysis, the NCD screening app was appreciated by ISHC health volunteers and supervisors, as an easier and more efficient way to report to higher levels, secure data, and strengthen relationships with relevant stakeholders, using tablets to connect to the internet and internet-based platforms to access information for self-learning and sharing to promote a healthy lifestyle as the strengths. The barriers and limitations reported by the respondents were a non–age-friendly app, incomplete translation of parts of the app into Vietnamese, some issues with the tablet’s display, lack of sharing of responsibilities among the health volunteers, and suboptimal involvement of the health sector; limited digital literacy among ISHC health volunteers. Recommendations are continuous capacity building, improving app issues, improving tablet issues, and involving relevant stakeholders or younger members in technology adoption to support older people.

**Conclusions:**

The implementation of the NCD screening app by ISHC volunteers can be an effective way to improve community-led NCD screening and increase the uptake of NCD prevention and management services at the primary health care level. However, our study has shown that some barriers need to be addressed to maximize the efficient use of the app by ISHC health volunteers to record, report, and manage the screening data.

## Introduction

Noncommunicable diseases (NCDs) have become a leading burden of disease, and accounted for 74% and 77% of all deaths globally and in Vietnam, respectively [1,2]. Older people are increasingly susceptible to NCDs and multimorbidity as they age [3] and Vietnam is among the most rapidly aging countries in the world [1-3]. The government is tackling an increased prevalence of NCDs [4,5], and one priority branch is to deal with hypertension and diabetes, which are recognized as having high prevalence, low percentage of awareness of their condition, and undertreatment [5,6]. In 2019, there were 11.8 million individuals with hypertension in Vietnam, while only 26.8% were under treatment [6]. It is estimated that 1 in every 20 Vietnamese adults has diabetes and more than 50% of patients with diabetes are unaware of their condition [7]. However, many obstacles still exist such as a lack of adequate resources and suboptimal policy implementation, hospitals are primarily responsible for providing NCD services, and provision of these services at primary health care (PHC) facilities is limited [4]. Therefore, the government has prioritized focusing on reaching more individuals for screening, diagnosis, and treatment of NCDs by strengthening the provision of services at the community level [4].

In this context, the research project “Scaling up non-communicable disease interventions in Southeast Asia” (SUNI-SEA) was implemented in Indonesia, Myanmar, and Vietnam. SUNI-SEA aimed to strengthen the quality and reach of NCD prevention and management services and to increase the linkage between the community and the PHC facilities [8].

In Vietnam, a component of community intervention in the SUNI-SEA project was implemented by 59 Intergenerational Self-Help Clubs (ISHCs), in which 295 ISHC health volunteers with a mean age of 65.3 years were working in Ninh Binh and Hai Phong provinces [8]. A detail of the ISHC model is presented in Multimedia Appendix 1 and widely documented [9-15]. In brief, ISHC is a community-based organization that has a dominant proportion of older members (about 70%). This model applies a comprehensive and inclusive approach that promotes healthy aging through a variety of interventions such as physical exercise, periodic health screenings, monthly monitoring of weight and blood pressure, health check-ups, and access to health insurance. During the project, ISHC health volunteers received initial training, health kits, and communication materials to conduct health promotion activities and NCD screening for risk factors of diabetes and hypertension with 6 tables of the screening process including (1) registration; (2) measuring waist circumference, height, weight, and BMI; (3) evaluating the Finnish Diabetes Risk Score (FINDRISC); (4) measuring blood pressure; (5) entering screening data into the NCD screening application; and (6) consultation (Multimedia Appendix 2). To maintain the sustainability and continuous strengthening of the capacity of ISHC health volunteers, 2 rounds of training, monitoring, and technical support trips were conducted [8]. Supervisors sometimes helped with data recording during screening activities. Members of ISHCs who were screened and classified as high risk of having hypertension or diabetes were advised to go to the PHC facility nearby for further diagnosis and treatment. Individuals who were diagnosed with an NCD in the PHC facility were treated and referred back to ISHCs after initial medical treatment, where ISHC health volunteers and club management boards helped to monitor their treatment adherence and promoted a healthy lifestyle (Multimedia Appendix 3).

There is mounting evidence of the relevance of eHealth apps in community health [16], including in Vietnam [17]. To further support ISHC health volunteers in capturing and using screening data, the SUNI-SEA project introduced a digital mobile NCD screening app in November 2021. This app was based on the open-source District Health Information Software, version 2 (DHIS2) tracker [18]. ISHC health volunteers used tablets to capture screening data, which were subsequently uploaded to a cloud server managed by HelpAge International in Vietnam (HAIV) once an internet connection was available. Training on using the tablet and the NCD screening app was integrated into the broader NCD prevention and management training. In addition, a tutorial video and user guidance for the app were provided to the ISHC health volunteers [19,20]. Due to the COVID-19 pandemic restrictions, the project implementation duration was approximately 1 year.

In the context of the digital era, eHealth interventions are supported for enhancing NCD management effectiveness, such as perspectives of clinical, behavioral changes, and service implementation outcomes. The recent reviews of eHealth interventions for NCD management in PHC settings of low- and middle-income countries found that eHealth interventions targeted health care providers or people with NCD mostly [16,17,21]. The studies, however, have not yet considered eHealth interventions for users who are volunteers, organizations, and older people in the community level. To fulfill this gap, this study aims to assess the strengths, barriers, and limitations of using DHIS2 on the tablet by ISHC health volunteers. Furthermore, it seeks to provide recommendations for the future use of digital NCD screening apps in community-based settings.

## Methods

### Study Design

This study uses a mixed methods observational approach with a convergent parallel design, aiming to achieve a comprehensive and multidimensional understanding of the implementation of a mobile health app in community-based organizations. In accordance with this design, quantitative data from the NCD screening app, collected by ISHC health volunteers in 2 provinces, were extracted and analyzed. These quantitative data facilitated the evaluation of ISHC health volunteers’ practices and the effectiveness of the app in supporting NCD prevention and management within ISHCs. Concurrently, qualitative research was conducted during and after the implementation of the screening app among ISHCs. These qualitative data provide deeper insights into user experiences and highlight the barriers and challenges encountered in integrating digital health apps into the ISHCs’ routine screening activities.

### Sample

In Vietnam, the SUNI-SEA project took place in the provinces of Ninh Binh and Hai Phong. Within these provinces, we implemented the intervention in 7 districts, of which 3 were urban and 4 were rural. In all 7 districts, the project was implemented in 44 purposely selected communes, which had 1 or more ISHCs, as well as a district health center and commune health station (CHS) provided both treatment and preventive services, which made up a sample of ISHCs (n=59) participating in the community-based intervention.

The ISHC health volunteers conducted 2 rounds of screenings in 2022, to which all members were invited. The first screening took place during the first half of 2022, and the second during the second half of 2022 with an interval of at least 6 months. In total 3463 individuals took part in the first screening and 3241 individuals took part in the second screening. Most of these individuals participated in both screenings (n=3222). Ultimately, data were collected from 3485 unique individuals among 59 ISHCs in these 2 provinces for the quantitative study.

**Figure 1:**
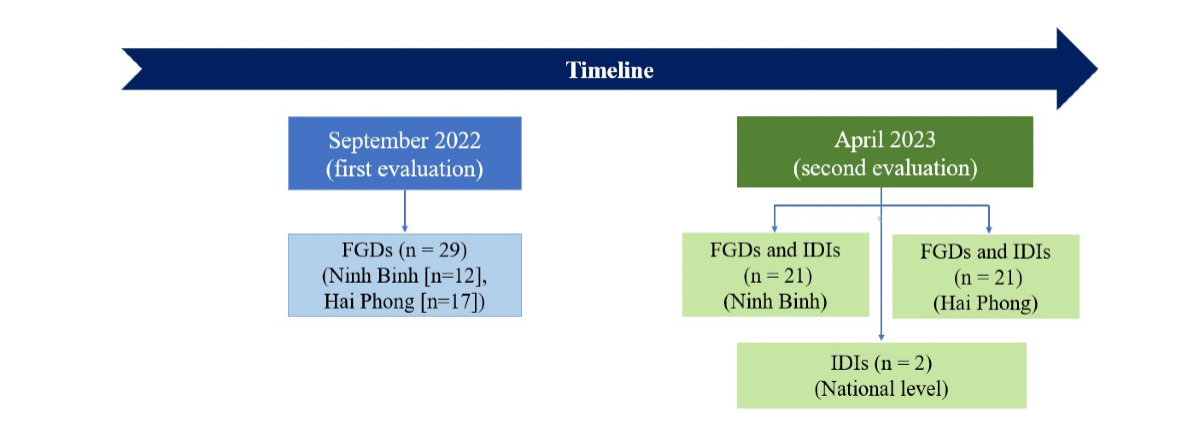
Timeline of data collection and study population. FGD: focus group discussion; IDI: in-depth interview.

In the qualitative study, we selected the ISHCs and the participants in screening purposively for inclusion in focus group discussions (FGDs) and in-depth interviews (IDIs), to achieve optimal diversity of users’ feedback. In total, 29 participants participated in FGDs in the first evaluation in September 2022 and 44 participants participated in FDGs and IDIs during the second evaluation in April 2023. In total, we had 70 unique participants; 3 individuals (each from a different ISHC) participated in both evaluations. We applied the following criteria for the selection of ISHCs and for individuals: (1) participants from ISHCs in both provinces, (2) participants from ISHCs in rural and urban areas, (3) participants from ISHCs who received previous financial and technical support from HAIV and ISHCs that did not receive previous support from HAIV (before the SUNI-SEA project), (4) ISHC participants who have experience entering screening data into the NCD screening application, and (5) participants who have not entered data into the NCD screening app before the first screening event in 2022. Figure 1 illustrates the sample of the qualitative study and the timeline of data collection.

To better understand the management aspects of the use of the NCD screening application and the potential for adoption and scaling up of the digital technology at the community level, various stakeholders were interviewed in the second evaluation, including representatives of the Association of the Elderly at the national, provincial, district and commune levels, and the CHS’s representatives. The representatives of commune and district stakeholders were based in the same areas as the selected ISHCs.

### Data Collection

The DHIS2 tracker software was used as a digital mobile NCD screening app for health volunteers in ISHCs to record and report data, which is available on the Google Play Store. To manage data security, HelpAge provided each ISHC with a log-in account to use and report data. The app was able to record data offline but to synchronize data to the main server, the device needed to connect to the internet. To ensure data protection, only the assigned HelpAge staff could log in and check the data of all ISHCs but from the ISHC level, users can review and check the data from their ISHC only. As guided by the project manual, screening was performed in a standardized manner, with predefined questions and measurements. The NCD screening app included questions about the demographics, that is, sex, age, marital status, place of living, and ISHC name. The survey included questions about previous diagnoses with an NCD, previous information about risk factors (eg, elevated glucose, overweight or obesity, and lifestyle issues), and the items from the FINDRISC questionnaire [22]. The ISHC health volunteers also measured the participants’ weight, height, waist circumference, and systolic and diastolic blood pressure; BMI was automatically calculated by the NCD screening app. The screening data were collected by trained ISHC health volunteers using tablets. However, often paper forms were used first, and the data were later entered into the screening app. All the data collected from 59 ISHCs were securely retrieved and included for further data analysis in the current study.

The first evaluation was conducted in September 2022 and the second was conducted in April 2023. During the first evaluation, the FGD guidelines and questionnaires were developed in English, translated into Vietnamese, tested, and adapted to the local participants’ backgrounds. The FGDs were conducted with selected ISHC health volunteers who met the inclusion criteria. Each FGD was conducted in a private place, sitting in a circle to ensure a friendly environment for the discussion with 1 facilitator and 1 note-taker who took minutes of the discussions. In the second evaluation, besides FGDs, IDIs were conducted. The IDIs were conducted individually and additional questions to obtain more in-depth information were asked. The interview guidelines were developed in English and translated into Vietnamese and adapted to the interviewees’ backgrounds. During the second evaluation, there were interviewers, note-takers, and interpreters (to interpret the questions and answers from English to Vietnamese and vice versa). To ensure accurate note-taking and reduce the loss of information missing, 2 note-takers were assigned to capture all answers during the interviews. FGDs and IDIs were not recorded in audio in both evaluations.

### Analysis

For the quantitative study, first, we provide a basic description of some of the demographic characteristics of our sample. To gain insight into the ISHC health volunteers’ use of the NCD screening app, we first assessed the completeness of the data. Finally, we checked whether there were potential errors in collected data by performing crosstabs of the item asking if an individual had previously been diagnosed with an NCD with subquestions specifying which NCD had been diagnosed, and we checked if plausible values were input for the anthropomorphic measures. Statistical analyses were performed using R (version 4.2.0; R Core Team).

The information received from the FGDs and IDIs of the qualitative study was manually coded and analyzed by identifying major themes, subthemes, codes, organizing the data, and interpreting the themes and ideas in the context. The codes were categorized based on the subquestions and purposes of the study within the following groups’ strengths, limitations or barriers, and recommendations for improving the use of app and further scaling up. The analysis was independently performed by 2 researchers (TTTN and GHN), who reconciled their findings afterward.

### Ethical Considerations

Ethical approval for the SUNI-SEA project in Vietnam was initially granted on November 13, 2019, by the Medical and Health Research Ethics Committee (approval number 019-485/DD-YTCC). Due to project disruptions caused by the COVID-19 outbreak, this approval was renewed on April 25, 2023 (approval number 196/2023/YTCC-HD3). In addition, an amendment for the use of quantitative data from the DHIS2 database was approved (298/2024/YTCC-HD3).

For privacy and confidentiality, all data were anonymized before analyzing. No compensation was provided to participants for taking part in this project. All participants received a full explanation of the objectives of the research and the interview process and were informed that they were free to opt out of participating. Informed consent was requested from each participant before the measurements were taken or interviews were conducted with 100% acceptance.

## Results

### Quantitative Results

#### Demographic Characteristics of the Screened Individuals

In total, 3485 individuals were screened. Of these, the majority were female (2642, 75.8% individuals). On average, the individuals screened were 65.3 (SD 10.1) years old. Most of the sample were either married (2968, 85.2% individuals) or widowed (406, 11.6% individuals). The remaining 3.2% of individuals were either single (84 individuals), divorced (14 individuals), or separated (13 individuals).

#### Completeness of the Data

Overall, the ISHC health volunteers filled in the required fields in the NCD screening app, often after first entering the data in paper-based records. The demographic data were recorded for all participants. The anthropomorphic data were nearly complete in all 6704 entries; diastolic blood pressure was not recorded for 1 individual during the second screening. The ISHC health volunteers generally filled in all the questions regarding having been previously diagnosed with an NCD. During the first screening, this was only missing in 0.4% (15/3463) of individuals. Similarly, during the second screening, this was only missing in 1.1% (36/3241) of individuals. The data concerning risk factors were not adequately recorded in 29.5% (1980/6704) of the screenings; 1156 entries were missing from the first screening and 824 entries were missing from the second screening. The FINDRISC items were mostly filled in. FINDRISC scores could not be calculated in 5.4% (186/3463) and 4.6% (150/3241) of participants from the first and second screenings, respectively. This was due to at least 1 item missing.

The quantitative results have pointed out that there was still a certain percentage of missing information from data entry by ISHC health volunteers. The qualitative results recorded several barriers and limitations in the use of the NCD screening app that help to explain the reasons.

#### Data Entry Errors

**Figure 2:**
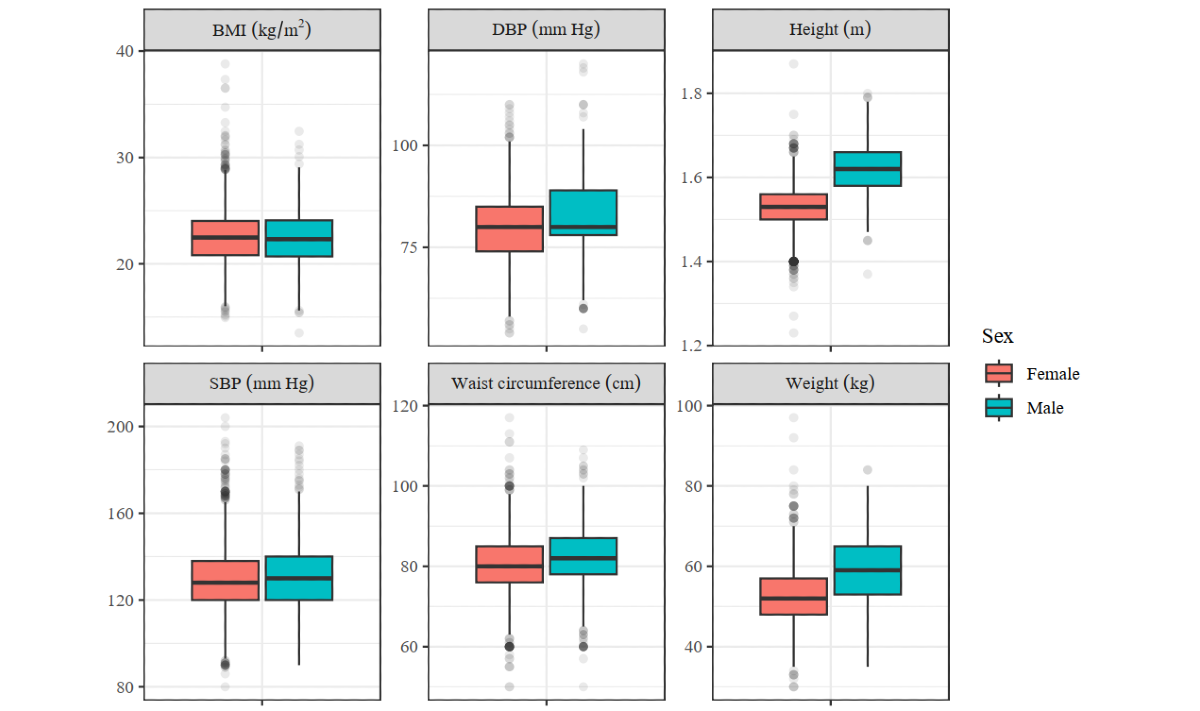
Distribution of the anthropometric measures from the first screening. DBP: diastolic blood pressure; SBP: systolic blood pressure.

**Figure 3:**
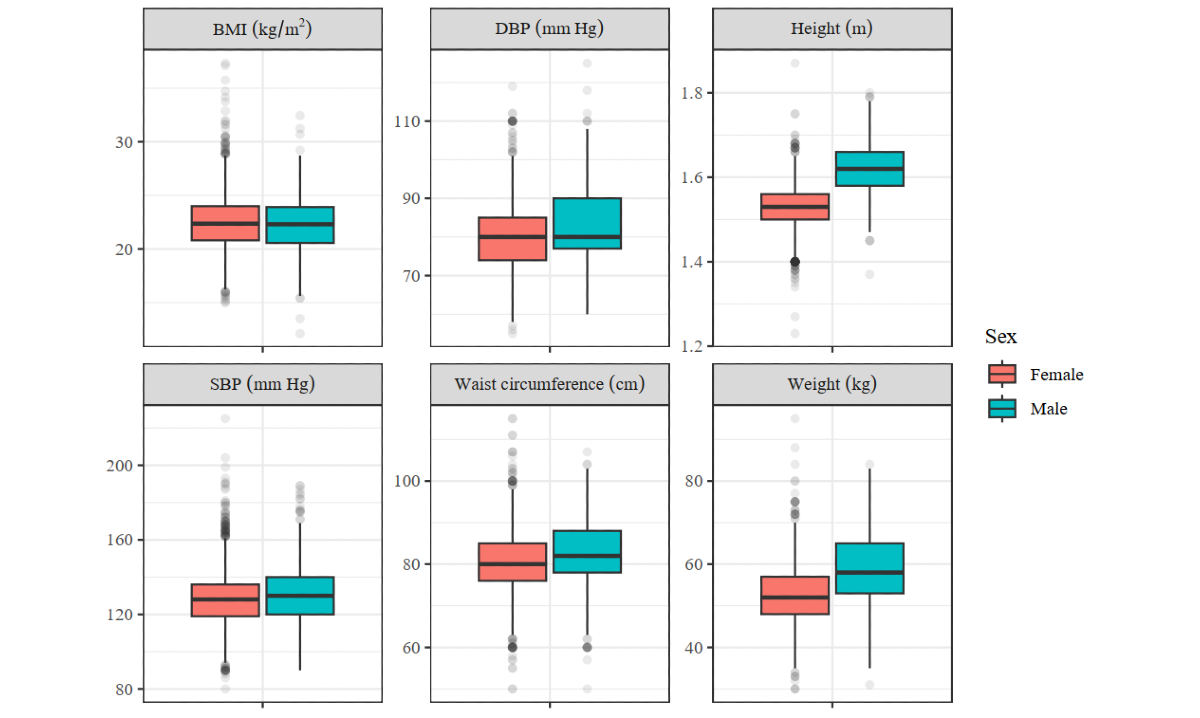
Distribution of the anthropometric measures from the second screening. DBP: diastolic blood pressure; SBP: systolic blood pressure.

When noting previous diagnoses of an NCD, in a few cases the ISHC health volunteers did not correctly record the diagnosis. This occurred 15 times during the first screening and 32 during the second screening. For the anthropometric data, we checked for plausible values by inspecting the distribution of these variables (Figures 2 and 3). Of the recorded values, 1.1% (71/6704) to 3% (204/6704) of values were outliers, for weight and height, respectively. However, at most 0.2% (11/6704) of the entries were defined as extreme outliers (a value that is 3 times the IQR away from the first or third quartile).

We checked the outliers individually and concluded that for height, weight abdominal circumference, and BMI, the outliers were generally plausible. Among older Asian females, few women with a low body weight are found. However, we suspect that in 5 cases errors were made when recording the height or weight, which resulted in a BMI below 15. The American Heart Association considers systolic blood pressure below 90 mm Hg and diastolic blood pressure below 60 mm Hg too low [23]. A total of 8 women had a systolic blood pressure below 90 mm Hg, and 11 women had a diastolic blood pressure below 60 mm Hg. Although it is clinically possible, these could also have been measuring errors.

### Qualitative Results

For the first evaluation, a total of 6 in-person FGDs were conducted in September 2022 in 13 ISHCs (9 ISHCs in Hai Phong and 4 ISHCs in Ninh Binh) with the participation of 29 ISHC health volunteers and club management board members. For the second evaluation, in 16 ISHCs (8 ISHCs in Ninh Binh and 8 ISHCs in Hai Phong) FGDs and IDIs were performed with a total of 44 interviewees.

#### Characteristics of Participants

The percentage of females in the first qualitative evaluation was 76% (22/29) and in the second evaluation, it was 57% (25/44). The average age during the first evaluation was 63.0 years and 64.2 years during the second evaluation. The percentage of people with college or university diplomas in the first evaluation was 14% (4/29) and 36% (16/44) during the second evaluation (Table 1).

**Table 1:** Detailed characteristics of participants.

Characteristics			Participants from the first evaluation (N=29), n (%)		Participants from the second evaluation (N=44), n (%)
**Sex**					
	Male	7 (24)		19 (43)	
	Female	22 (76)		25 (57)	
Average age, mean (SD)			63.0 (9.7)		64.2 (10.2)
**Geographic area**					
	Rural	19 (66)		22 (50)	
	Urban	10 (35)		22 (50)	
**Education status**					
	Secondary school	6 (21)		6 (14)	
	High school	19 (65)		22 (50)	
	College or university	4 (14)		16 (36)	

#### Findings

In evaluating the usability of the NCD screening app for ISHC health volunteers and ISHCs, 3 main themes were identified through manual coding, including strengths in the use of the NCD screening app, barriers and limitations in the use of NCD screening app, and recommendations for improving the use of NCD screening app and further scaling up. This resulted in a wide range of sometimes contradicting opinions, both positive and negative. The answers per theme are listed in Table 2. The full table of answers with themes, subthemes, and codes is described in Multimedia Appendix 4. Interviewees reflected on the technical issues of the app, on the capacity of volunteers using it, and on organizational and strategic issues of electronic data collection. Besides giving positive and negative opinions they made concrete suggestions for improvement. The frequency of responses is provided combined with quotes in Tables 3-5. This helps to get a better insight into the importance of some remarks, and more context of comments provided during FGDs and IDIs.

**Table 2:** Categories of findings and answers from interviewees.

Themes	Subthemes
Strengths in the use of the NCD^a^ screening app	Advanced app functions Building capacity for management boards, ISHC^b^ health volunteers, and members Improving the health of ISHC members Increasing reputation for club management board members and ISHC health volunteers with their club members Strengthening relationships with stakeholders Helping the implementation of the national ISHC model Storing photos of ISHC’s activities on the tablet Others
Barriers and limitations in the use of the NCD screening app	App issues Limited capacity of ISHC health volunteers/ older people in technology Tablet issues Irregular technical support after initial training due to COVID-19 restrictions Others
Recommendations for improving the use of the NCD screening app and further scaling up	Continuous capacity building Improving app issues Improving tablet issues Involving relevant stakeholders Involving younger members in tablet use or technology adoption to support older people Others

^a^NCD: noncommunicable disease.

^b^ISHC: Intergenerational Self-Help Clubs.

**Table 3:** Quotes on strengths in the use of the noncommunicable disease screening app.

Strength	Quote	Participant description
Advanced app functions	“Using a tablet and the NCD^a^ screening application to report data helps me not to have to report by paper forms, just enter data into the application and then press sync function then data is sent very quickly, saving money and time.”	Female, ISHC^b^ health volunteer, Hai Phong
Building capacity for management boards, ISHC health volunteers, and members	“In addition, my club also uses a tablet connecting to Zalo groups to receive information and read newspapers to raise awareness, and access information and technology.”	Female, chairperson and ISHC health volunteer, Ninh Binh
Improving the health of ISHC members	“…. but the tablet is also used by the club management board to connect to the network with songs and performances for physical exercises for all club members. That’s good for us.”	Female, chairperson and ISHC health volunteer, Ninh Binh
Increasing reputation for club management board members and ISHC health volunteers with their club members	“… I feel more confident. Club members trust me, ISHC health volunteers, and the Club Management Board more because they saw us using the tablet phone and the NCD screening application to record their screen data. We can do the same as younger people. They (club members) feel happy and secure.”	Female, chairperson, Hai Phong

^a^NCD: noncommunicable disease.

^b^ISHC: Intergenerational Self-Help Clubs.

**Table 4:** Quotes on barriers and limitations in the use of the noncommunicable disease screening app.

Barrier and limitation	Quote	Participant description
App issues	“Some parts of the software are still in English, so it is difficult to remember. After the training, when we go home and practice, we see the English parts then we would forget everything.”	Female, ISHC^a^ health volunteer, Hai Phong
Limited capacity of ISHC health volunteers or older people in technology	“I feel difficult and confused because the application is new and I’m old.”	Female, ISHC health volunteer, Hai Phong
Limited capacity of ISHC health volunteers or older people in technology	“I’m afraid that when I press the wrong button then it will log out and difficult to log in back.”	Female, chairperson and ISHC health volunteer, Ninh Binh
Tablet issues	“Letters and keyboard in the tablet phone are small and my eyesight is poor, so I often make a mess.”	Female, ISHC health volunteer, Ninh Binh

^a^ISHC: Intergenerational Self-Help Clubs.

**Table 5:** Quotes on the recommendations.

Recommendation	Quote	Participant description
Continuous capacity building	“Training, refresh training, and technical support are necessary for us to use app fluently.”	Female, chairperson and ISHC^a^ health volunteer, Ninh Binh
Continuous capacity building	“Tutorial video is useful.”	Female, ISHC health volunteer, Hai Phong
Improving app issues	“Converting language from English to Vietnamese in few parts so we can remember better.”	Male, ISHC health volunteer, Hai Phong
Involving younger members in tablet use or technology adoption to support older people	“We’re old, the application is new, we need support from younger members.”	Male, chairperson, Hai Phong

^a^ISHC: Intergenerational Self-Help Clubs.

### Strengths in the Use of the Noncommunicable Disease Screening App for Health Volunteers and Intergenerational Self-Help Clubs

The majority of the answers focused on advanced functions of the NCD screening app (eg, better data storage, faster and easier reporting to HAIV, more time efficiency, and saving money; 112 responses). The following are building capacity for ISHC health volunteers in using technology (26 responses), improving the health of ISHC members through connecting the internet for learning skills and doing exercise (26 responses), increasing the reputation of club management board members and ISHC health volunteers with their club members (13 responses), strengthening relationships with stakeholders (6 responses), and others (9 responses). These advantages could explain the great results of data entry by ISHC health volunteers.

### Barriers and Limitations in the Use of the Noncommunicable Disease Screening App for Health Volunteers and Intergenerational Self-Help Clubs

Most of the answers focused on app issues (eg, a few parts of the app are still in English, typographical errors, bugs or errors, few parts of the display screen are covered by the keyboard, many steps to register screening data, and hard to add new members; 67 responses) and limited capacity of ISHC health volunteers or older people in technology (67 responses). The following includes tablet issues (small font size; 13 responses), irregular technical support after initial training due to COVID-19 restrictions (6 responses), and other issues (eg, Wi-Fi connection problems and changes in the personnel who is in charge of the tablet; 15 responses). These barriers and limitations could be the reasons of certain data that are missing and few errors that are recorded in quantitative results.

### Recommendations for Improving the Use of the Noncommunicable Disease Screening App

The main recommendations recorded from the qualitative analysis are continuous capacity building (34 responses), improving app issues (27 responses), and improving tablet issues (24 responses). The following includes involving relevant stakeholders (7 responses), involving younger members in tablet use or technology adoption to support older people (7 responses), and others (13 responses; eg, financial support, more measurement devices for the screening, and supporting internet connection or internet packages).

## Discussion

### Principal Results

This study aimed to evaluate the strengths, limitations, opportunities, and barriers of the NCD screening app used by ISHC health volunteers, and to provide recommendations for future use of the digital NCD screening app in community settings. Quantitative analysis revealed that nearly one-third of the risk factor data were missing and there were a few errors in data entry. However, the NCD screening app demonstrated significant advantages, including easier data storage, better space and time savings, more efficient submissions of reports to higher levels, and improved security compared with paper forms. The key barriers to using the app included the advanced age of some volunteers, lack of previous experience, and low digital literacy among ISHC health volunteers.

The quantitative data analysis showed that the ISHC volunteers generally filled in all required fields and there were few obvious errors in the data entry. The main source of missing data was the recording of risk factors. As indicated by FGDs with health volunteers, a possible explanation for this is that ISHC health volunteers may have only checked the boxes when a risk factor had been identified and skipped the question if no risk factors were identified. This could be attributed to a misunderstanding of how to fill in the questionnaire, which was occasionally noted during the training sessions by HAIV staff. Other reasons, identified as key findings of the qualitative evaluation of the use of the NCD screening app, include the limited digital literacy of older health volunteers, poor vision, the small keyboard, errors in the app, and irregular technical support during the COVID-19 pandemic. These factors likely contributed to data entry mistakes or unintentional data omissions by health volunteers.

It is important to note here that, initially, in most ISHCs, data were first entered manually in registers and later entered electronically in the tablet. The ISHC health volunteers did not want to slow down the screening process, as they were more familiar with paper-based systems. Supervisors in several instances assisted with entering the data. There is no record of the frequency of assistance by supervisors. Therefore, our finding that health volunteers were able to enter the screening data completely and correctly may be influenced by the support of supervisors during the screening.

The percentage of females who participated in the qualitative evaluations was much higher in the first evaluation (22/29, 76%) compared with the second evaluation (25/44, 57%) and the percentage of people with college or university diplomas in the second evaluation (16/44, 36%) was higher than in the first evaluation (4/29, 14%). This is partly to be expected as the participants of the first evaluation mostly consisted of ISHC health volunteers, club chairpersons (most of the club members were female [70%, as per ISHC’s regulation]), and community members with lower levels of education. The second evaluation included more stakeholders from higher levels including representatives from the Association of the Elderly at all levels and representatives from CHSs were included. It is aligned with data from the quantitative survey with 75.8% (2642/3485) of screened people being female.

The qualitative analysis has shown that adoption of digital technology at the community level brings many benefits to health volunteers, ISHCs, and the local Association of the Elderly. ISHC health volunteers found that the NCD screening app helps them more easily store data, saves both space and time, facilitates sending reports to higher levels, is more secure compared with paper forms, and automatically calculates scores. The analysis of data using the dashboard provides faster in-depth information. The advantages of the NCD screening app make the work easier for ISHC health volunteers, which is aligned with previous studies [24,25]. The Vietnamese government has been moving toward increased digital transformation, particularly digital transformation in health care [23,26,27]; therefore, the use of digital technology at the community level is aligned with the country’s strategy, helping health volunteers and ISHCs adopt IT knowledge which is vital so that they are not left behind [28]. Furthermore, the analysis of the data collected with the NCD screening app increases awareness of NCD symptoms leading to better self-care, more frequent visits to the health facilities and potentially contributing to improved overall health of ISHC members.

The most frequently mentioned barrier during the interviews was the health volunteers’ old age with an average age of 63.8 years. Furthermore, many ISHC health volunteers and club management boards lacked experience with tablets, smartphones, the internet, and other IT knowledge [25,29]. As shared by the ISHC health volunteers during FGDs due to their perceived low digital literacy, they initially preferred to continue using paper-based records. This can be partially explained by the quantitative findings which indicated some errors or missing data. Although HAIV provided additional training, support, and e-learning tools, these efforts did not always seem sufficient.

A notable difference was observed between rural and urban ISHCs in their experience with the NCD screening app. Urban health volunteers and ISHCs more frequently mentioned the app’s strengths likely due to their greater familiarity with smartphones and internet. In contrast, rural ISHCs reported more limitations. Learning IT was not a priority for many rural health volunteers who were primarily focused on agriculture, fishing, and family care. Some rural ISHC health volunteers mentioned that they had never used a smartphone. Digital literacy remains a significant challenge in the introduction of the community-based app, requiring more time and training than is typically allocated in projects [30]. Once the health volunteers become confident in using the NCD screening app, they can enter data on the spot and transition away from paper registers.

In addition, several issues related to the screening app were recorded, such as a non–age-friendly app, confusing display, incorrect translation of medical terminology, language barriers knowledge [25], small font size on the tablet, and so on. Furthermore, internet-based software requires an internet connection to upload the data to the server, which was a problem for some rural ISHC health volunteers with poor internet connections. These findings are similar to those of a study in Bangladesh [31].

The short implementation time due to the COVID-19 pandemic was recorded as one of the barriers where participants mentioned irregular technical support after the initial training for ISHC health volunteers and club management boards and a pause in project activities due to pandemic restrictions.

Health systems in low and middle-income countries are slow in adopting technology at the community level [24,25,32,33]. This may lead to limitations in sharing and using data at higher levels, especially the use of data for planning and policy-making [31]. These findings are aligned with our study because interviewees from the national level mentioned that the NCD screening app we tested cannot be easily used in existing PHC systems.

An additional function of the NCD screening app that can be used for ISHCs in Vietnam is the monthly report. In ISHCs, where the functions of the app were better used, it helped club management boards save time and space to store reports, making reports easier and faster to be sent to higher levels of the Association of the Elderly. The ISHCs can use the dashboards in the app for improved monitoring and planning of activities. In the long run, expanding the NCD screening app with monthly reporting indicators can completely replace paper forms and reduce the workload for management boards and ISHCs [29].

### Study Strengths and Limitations

A strength of this research is the use of convergent parallel design, combining quantitative data with detailed insights from qualitative data. With the use of semistructured interviews or FGDs, the experience of the users of the app was examined in depth.

There are a few limitations that should be discussed. With parallel implementation, some qualitative findings cannot explain or answer questions and issues from quantitative findings. In some instances, corrections of wrong data entries may have been made immediately after the screening sessions, when data were transferred from paper-based to digital systems. Furthermore, supervisors in several instances assisted in entering data. This may have led to a more positive picture of data quality.

In the first round of qualitative evaluation, there was only 1 note-taker and no data cross-check, which may have led to data loss. Due to the presence of HAIV staff, representatives of the Provincial Association of the Elderly, and the local Association of the Elderly, the interviewees may have had a bias in their responses in both evaluation rounds. In addition, in the second evaluation round data may have been lost during the translation process.

### Implications

Applying the NCD screening app in NCD intervention through ISHCs in Ninh Binh and Hai Phong in Vietnam in the SUNI-SEA project has provided multiple lessons for the use of digital technology at the community level.

To strengthen the use of the tablet NCD screening app among health volunteers or ISHCs, simplifying the app, fixing errors, converting English text into Vietnamese, and redesigning it to be an age-friendly app that is easier for older people to use is necessary. Involving users, especially end users (local people or older people), in the design stage would help the design to be more user-friendly and aligned to the needs of the community and older people.

Providing continuous capacity-building opportunities, longer implementation time of the project, longer team support and commitment, allotting more time for practice sessions, adding separate general internet technology sessions for ISHC health volunteers and older people, provision of regular technical support, and recruiting younger members to join ISHCs and health volunteer groups are recommended for ISHC health volunteers and old-aged–groups. These recommendations from our study are consistent with the suggestions documented in other studies, which suggest that additional training and ongoing technical support should be continued to improve knowledge and efficiency with any update of the DHIS2 tracker [25,29,33].

To leverage the linkage between ISHC or the Association of the Elderly and the health sector, the use of screening data by health sectors and authorities, integrating the app into the existing health information system is recommended to provide an official referral system and use of the data for planning and decision-making. DHIS2 tracker is an open-source software with good interoperability, and linking this community-based information system to the PHC system should be the next step.

For the health sector, it would be beneficial if the referral mechanism is systematic and official from ISHC to PHCs and vice versa. With this mechanism, PHCs can diagnose NCDs earlier, provide proper treatment for patients, and better manage NCDs at the community level. Adopting technology at the community level is also consistent with the health system strategy and digital transformation of the Vietnamese government [26,27], while maximizing the use of local resources and support from health volunteers and ISHCs as a contribution to the NCD prevention and management programs in the context where there is a lack of resources within the health sector [34].

Adopting the NCD screening app in ISHCs is to help health volunteers and communities have an easier way and more effective to track and manage NCD screening data, especially with high-risk members. Using the NCD screening app can generate quality data that can be used for analysis, advocacy, and decision-making for the health sector in NCD programs across the country.

### Conclusion

Our findings show that the NCD screening app brings a lot of benefits for health volunteers and ISHCs, where all ISHC health volunteers and club management boards are voluntarily donating their time and ability to contribute to NCD prevention and management at the community level, including managing screening data for their club members.

Adopting technology for NCD prevention and management at the community level is not an easy process. It requires the involvement of several stakeholders from the grassroots to higher levels in order to maintain sustainability and effective data use in NCD prevention and management, especially for the health sector in strategic planning and decision-making at higher levels.

In summary, the implementation of the NCD screening app at the community level in Vietnam can be an effective way to improve health screening for ISHCs and community members and NCD prevention and management for the health sector. However, our study has shown that there are still various barriers that limit the ISHC health volunteers who use the NCD screening app to record, report, and manage screening data in the app correctly.

## Appendix

Multimedia Appendix 1 Intergenerational Self-help Club Model.

Multimedia Appendix 2 Noncommunicable disease screening process.

Multimedia Appendix 3 The synergy approach of the SUNI-SEA (Scaling up non-communicable disease interventions in Southeast Asia) project.

Multimedia Appendix 4 Categories of findings and answers from interviewees (full Table 2).

## Abbreviations

CHS: commune health station
DHIS2: District Health Information Software, version 2
FGD: focus group discussion
FINDRISC: Finnish Diabetes Risk Score
HAIV: HelpAge International in Vietnam
IDI: in-depth interview
ISHC: Intergenerational Self-Help Club
NCD: noncommunicable disease
PHC: primary health care
SUNI-SEA: Scaling up non-communicable disease interventions in Southeast Asia
