# Supplementary figures and images for: Challenges and Opportunities in Digital Screening for Hypertension and Diabetes Among Community Groups of Older Adults in Vietnam: Mixed Methods Study
Source: J Med Internet Res. 2024 Dec 2;26:e54127. doi: 10.2196/54127 (PMC11650079; doi:10.2196/54127)

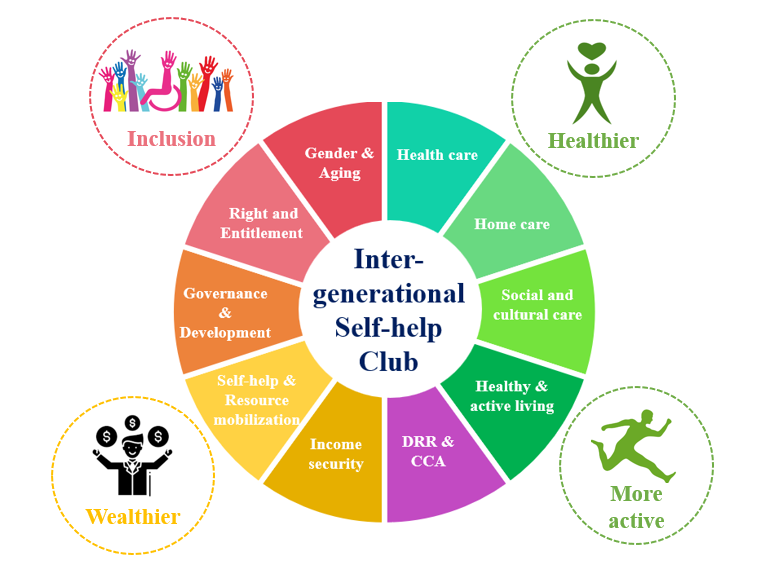

Supplement: Multimedia Appendix 1 [file jmir_v26i1e54127_app1.png]

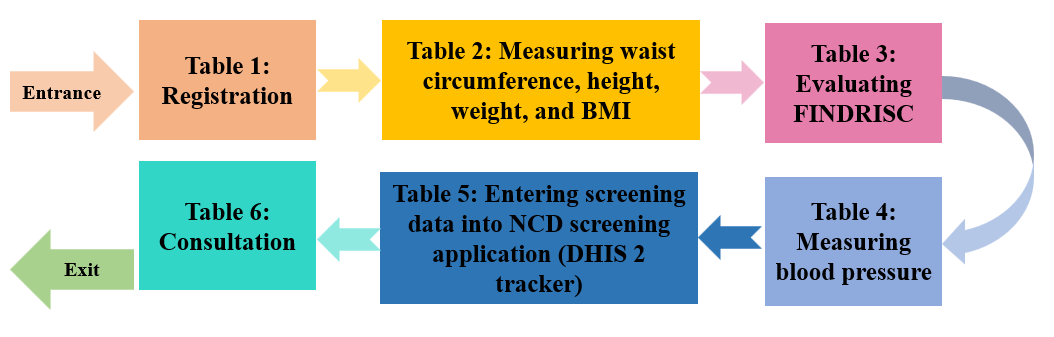

Supplement: Multimedia Appendix 2 [file jmir_v26i1e54127_app2.png]

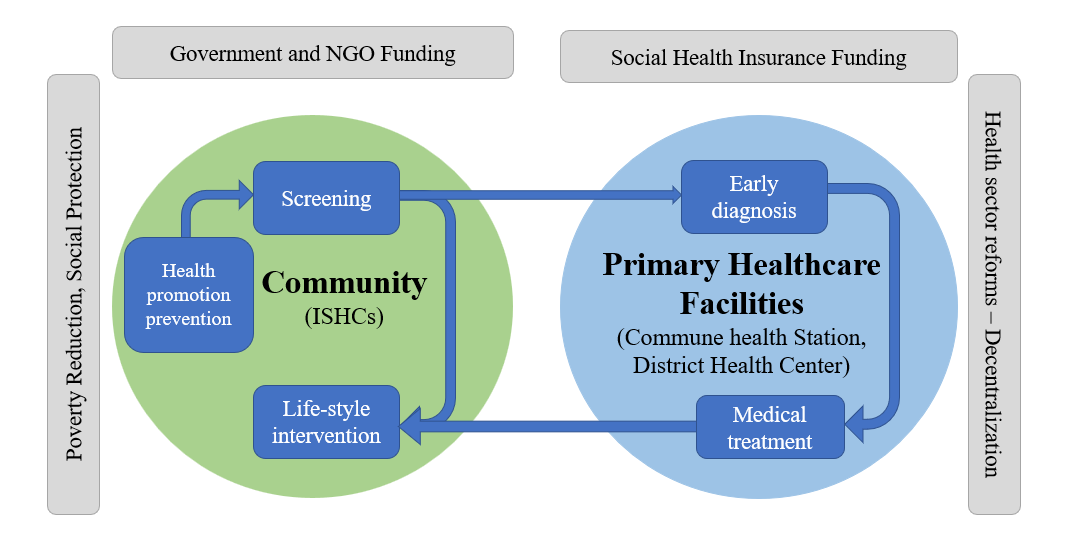

Supplement: Multimedia Appendix 3 [file jmir_v26i1e54127_app3.png]
