# Supplementary material for: Challenges and Opportunities in Digital Screening for Hypertension and Diabetes Among Community Groups of Older Adults in Vietnam: Mixed Methods Study
Source: J Med Internet Res. 2024 Dec 2;26:e54127. doi: 10.2196/54127 (PMC11650079; doi:10.2196/54127)
Supplement: Multimedia Appendix 4 [file jmir_v26i1e54127_app4.docx]

| Themes | Sub-themes | Codes |
| --- | --- | --- |
| Strengths in the use of NCD screening application | Advanced application functions | Better storage of screening data (saving space) |
|  |  | Fast and complete data management online |
|  |  | Easier reporting to Associations of the Elderly and HAIV |
|  |  | More time efficient when data are entered on the spot |
|  |  | No travel costs/saving money |
|  |  | Reducing workload (less work, making work easier) |
|  |  | Fewer mistakes compared with paper forms |
|  |  | Automatic calculation of BMI and FINDRISC scores with outcome in color and alert notes if missing data or high-risk scores |
|  |  | Easier to compare data with previous screening |
|  |  | Friendly application |
|  |  | Easier correcting mistakes |
|  |  | Smaller weight compared to registers |
|  | Building capacity for management boards, ISHC health volunteers, and members | Increasing technology knowledge |
|  |  | Raising awareness of NCDs and healthcare |
|  |  | Building capacity for management boards and ISHC health volunteers in NCD screening |
|  |  | Being more careful |
|  | Improving the health of ISHC members | Overall improving the health of ISHC members |
|  |  | Using the tablet to connect with online music for the group cultural performances and physical exercise |
|  | Increasing reputation for club management board members and ISHC health volunteers with their club members | |
|  | Strengthening relationships with stakeholders | Collaborating with the health sector |
|  |  | Strengthening relationships with club members |
|  |  | Connecting with other ISHCs via mutual groups/social networks |
|  | Others | Overall useful application |
|  |  | Helping the implementation of the national ISHC model |
|  |  | Storing photos of ISHC’s activities on the tablet |
| Barriers and limitations in the use of NCD screening application | Application issues | The application is not fully developed:   - A few parts of the text are in English - Typos in the Vietnamese text - Bugs/errors in the application - Difficult to differentiate between the titles of sections and the text of questions - Part of the screen is covered by the keyboard |
|  |  | Non-aged-friendly application/confusing display |
|  |  | Many steps to register screening data |
|  |  | Not time efficient when data first entered on paper |
|  |  | Different order of filling in data compared with paper forms |
|  |  | Hard to add new members |
|  |  | No quick fix of the bugs by the technology company |
|  |  | Limited availability (on Android phones/tablets only) |
|  | Limited capacity of ISHC health volunteers/ older people in technology | Lack of IT knowledge/unfamiliarity with technology |
|  |  | Forgetting steps to use the application after a while |
|  |  | Poor vision |
|  |  | Filled-in data is incomplete, incorrect, or filled-in the wrong function |
|  |  | Fear of breaking or making a mess |
|  |  | Adding a member twice |
|  |  | Taking much time to provide more training due to limited capacity |
|  | Tablet issues | Small font size |
|  |  | One person is responsible for the tablet |
|  | Irregular technical support after initial training due to COVID-19 restrictions | |
|  | Others | WIFI/problems connecting to the internet |
|  |  | Changing in personnel |
|  |  | Difference in capacity among ISHCs |
|  |  | The health sector does not have access to the application |
|  |  | Data is not analyzed by health volunteers |
| Recommendations for improving the use of the âzNCD screening application and further scaling up | Continuous capacity building | More training/technical support about the application and technology |
|  |  | More instruction or video on the use of the application |
|  |  | Members need to put more effort into understanding the application |
|  |  | More practice |
|  |  | Knowledge of the screening process will help to enter data into the application |
|  | Improving application issues | Converting full text into Vietnamese |
|  |  | Additional screening for mental disorders |
|  |  | Including the ISHC health volunteers/users in the developing process |
|  |  | Simplifying application/display (shorten steps) |
|  |  | Assigning copyright to local companies/ implementers |
|  | Improving tablet issues | More tablets available |
|  |  | Sharing responsibility for the tablet |
|  | Involving relevant stakeholders | Involving the health sector |
|  |  | Implementing the application at higher levels |
|  | Involving younger members in tablet use/ technology adoption to support older people | |
|  | Others | Financial support |
|  |  | More measurement devices for the screening |
|  |  | Supporting internet connection/internet packages |
|  |  | Using the application also for monthly check-ups |
|  |  | Having a direct contact for help |
